# Supplementary material for: “The algorithm will screw you”: Blame, social actors and the 2020 A Level results algorithm on Twitter
Source: PLoS One. 2023 Jul 26;18(7):e0288662. doi: 10.1371/journal.pone.0288662 (PMC10370707; doi:10.1371/journal.pone.0288662)
Supplement: Attachment — Submitted filename: PLOS One Rebuttal Letter May 23.docx [file pone.0288662.s002.docx]

School of Computer Science

University of Nottingham

Wollaton Road

Nottingham

Nottinghamshire

United Kingdom

NG8 1BB

Friday 12th May 2023

Dear Editors,

We, the authors, would like to thank both reviewers for their insightful feedback on our manuscript. Each suggestion was thought-provoking and helpful. As a result, we have edited the manuscript to address their comments.

Please see the table below that indicates our response to each suggested change.

We now believe that this manuscript is suitable for publication in PLOS One.

Yours faithfully,

Dan Heaton

On behalf of all authors

| **Comment** | **Page(s)** | **Action** |
| --- | --- | --- |
| The dataset of tweets is almost three-year-old. What is the significance of analyzing this old dataset? Do your conclusions still hold in 2023? | 20 | We thank the reviewer for this comment. Whilst the disruption of the pandemic is coming to an end, this paper gives insight into how members of the public may react to future decision-making algorithm interventions. We have included this as a statement in the conclusion. |
| A better explanation and subsequent implications are needed for the drop in the VADER line around August 2020 in Figure 1. | 4 | More detail has been added here – this is most likely caused by ‘mutant’ holding negative sentiment score, combined with an increase in the number of negative responses. |
| Please explain what you mean by the agency. Do social actors always act as a social agency? A literature review on agency and situating your study against past research will be useful. | 7-8 | Thank you for this suggestion. We define agency on page 7 of the manuscript. We have expanded on the referenced literature on page 9 relating to using agency alongside CL and CDA to explore social media discourses. |
| How do different social actors interact with each other, and what are the implications of their interaction for your study? | 20 | We have considered this carefully and have concluded that the aim of the paper is to look at how the social actors are represented, rather than how they interact with one another. We have, instead, offered a future work suggestion for exploring this. |
| The authors state “there is a research gap regarding public views expressed on Twitter […]”. I agree, but why does this need to be addressed? The following text does not explain this. | 1-2 | Further explanation and detail has been added after this sentence to explain how using social media data can add to the bigger picture of the public’s response to the algorithm. |
| The rest of the introduction is very much about the chosen methodological approach. This is understandable. However, to underline the practical relevance of the authors’ approach, I would suggest spending some more time on contextualizing the methods in the wider, practical discourse. I understand that this is what the next section does. I think it would be an alternative option to already mention more about this earlier. | 2-3 | We have added a sentence to make reference to contextualising the methods in the wider, practical discourse and alluded to how these will be explored later in the paper. |
| However, towards the end of the ‘Context of the Algorithm’ section, I think the authors should consider referring back to why and how their methods are going to add more information about the discourse. | 3 | We agree and have added a short paragraph explaining this. |
| Moreover, the authors state “yet limited research into how social media users reacted to 101 the scandal, thus providing motivation for our research”. Again, I agree. But for the purpose of the paper, I think the authors should spend more time in explaining why this is important and what additional insights it can provide. The next section does this – I just think that the transitions could be improved and made a bit more fluent. | 3 | This has been addressed in the additional paragraph also. |
| “Using CL and CDA, underpinned by SAR, it will be possible to ultimately contribute to filling the gap previously identified in the literature.“ Why and how? | 4 | This sentence has been extended to add further clarity. |
| NLP-Based Computational Linguistics to Examine Social Media: It seems that this section already provides results from the collected data. However, this is really not clear to me. Overall, this section is difficult to read and follow. | 4-5 | More discourse markers have been added to ensure clarity in this section. |
| Using Critical Discourse Analysis to Examine Social Media: I like this section and how the authors describe how this approach can adhere to the gaps of the other approach. Yet, the link to social media and Twitter is rather short and I would like to suggest that the authors spend more time on expanding the paragraph on page 7, lines 277-287. | 7 | Thank you for this comment. We have expanded this section with more specific findings from these case studies to illustrate the depth of understanding that CDA can uncover. |
| Research Gap: I think Social Actor Representation (SAR) and Social Action Theory (SAT) need to be mentioned earlier in the manuscript. This seems to be crucial for the paper. But only surface just before the Methods section. Right now, the link to the method and why it is important are too short and sometimes just constructed with one sentence. | 8 | We thank the reviewer for this observation and suggestion. Upon reflection, we have kept the main part of SAR and SAT in this section. However, we have introduced and provides information about these concepts earlier in order to foreground their importance in our work. |
| Method: Good. I just think that Table 1 is not positioned well in this section. | 10 | Agreed. This has been adjusted so it is in section 4. |
| “Based on this first list, four potential social 379 actors (the algorithm, Ofqual, the government and students) were investigated through 380 the examination of collocational strength and CDA.” I am struggling a bit to consider “the algorithm” as a social actor. I think I know what the authors are referring to, but maybe they could spend some more time making an argument that this link can be made. The discussion touches upon this. But, in my opinion, it might be a bit late then. | 10 | Additional rationale has been included here: as all of these words are nouns that can be presented actively in a grammatical construction, they are all capable of being a social actor. |
| The Algorithm: This is an interesting section. However, I think it would be really beneficial to add another Figure that shows the frequency of the collocations across time. I think it would add another very valuable layer to the description in the text. Similarly, while the next two sections are equally interesting, I wonder whether the authors could consider a more visual representation of their findings as they have a clear timeline going through the analyses. | Figures | Linked scatter diagrams – grouped on three day intervals – have been inserted to show the trajectories of the LogDice scores over time. These have been split into part A and part B to prevent cluttered figures. |
| Limitations and Future Work: I was a bit surprised not to see any reference to Social Network Analyses, which particularly in combination with CL is becoming more common in research. Additionally, while I understand the authors’ criticism of sentiment analyses, I think that it would have been interesting to more carefully combine this with their CL approach of collocations and POS. | 18-19 | We have considered this and feel that Social Network Analyses would be an example of future work. Thus, we have included reference to this in this section. |
| Conclusion: The methodological conclusion is understandable. The overall conclusion of “blaming a social actor” is of course more difficult, but also less clear in the description. | 20 | Discourse markers have been added to make the conclusion clearer: all three of the social actors explored are blameworthy, although it is not possible to confirm which one was blamed the most. |
